# HIV-1 Vif N-terminal Motif is required for recruitment of Cul5 to Suppress APOBEC3

**DOI:** 10.1186/1742-4690-11-4

**Published:** 2014-01-14

**Authors:** Sean L Evans, Arne Schön, Qimeng Gao, Xue Han, Xiaohong Zhou, Ernesto Freire, Xiao-Fang Yu

**Affiliations:** 1 Department of Molecular Microbiology and Immunology, Johns Hopkins Bloomberg School of Public Health, 615 N. Wolfe Street, Baltimore, MD 21205, USA; 2 Department of Biology, Johns Hopkins University, Baltimore, MD USA; 3 School of Life Sciences, Tianjin University, Tianjin, China; 4 Institute of Virology and AIDS Research, First Hospital of Jilin University, Changchun, Jilin Province, China

## Abstract

**Background:**

HIV-1 Vif promotes the degradation of host anti-retroviral factor family, APOBEC3 proteins via the recruitment of a multi-subunit E3 ubiquitin ligase complex. The complex is composed of a scaffold protein, Cullin 5 (Cul5), RING-box protein (Rbx), a SOCS box binding protein complex, Elongins B/C (Elo B/C), as well as newly identified host co-factor, core binding factor beta (CBF-β). Cul5 has previously been shown to bind amino acids within an HCCH domain as well as a PPLP motif at the C–terminus of Vif; however, it is unclear whether Cul5 binding requires additional regions of the Vif polypeptide.

**Results:**

Here, we provide evidence that an amino terminal region of full length Vif is necessary for the Vif-Cul5 interaction. Single alanine replacement of select amino acids spanning residues 25–30 (^25^VXHXMY^30^) reduced the ability for Vif to bind Cul5, but not CBF-β or Elo B/C in pull-down experiments. In addition, recombinant Vif mutants had a reduced binding affinity for Cul5 compared to wild-type as measured by isothermal titration calorimetry. N-terminal mutants that demonstrated reduced Cul5 binding were also unable to degrade APOBEC3G as well as APOBEC3F and were unable to restore HIV infectivity, in the presence of APOBEC3G. Although the Vif N-terminal amino acids were necessary for Cul5 interaction, the mutation of each residue to alanine induced a change in the secondary structure of the Vif-CBF-β-Elo B/C complex as suggested by results from circular dichroism spectroscopy and size-exclusion chromatography experiments. Surprisingly, the replacement of His108 to alanine also contributed to the Vif structure. Thus, it is unclear whether the amino acids contribute to a direct interaction with Cul5 or whether the amino acids are responsible for the structural organization of the Vif protein that promotes Cul5 binding.

**Conclusions:**

Taken together, we propose a novel Vif N-terminal motif that is responsible for Vif recruitment of Cul5. Motifs in Vif that are absent from cellular proteins represent attractive targets for future HIV pharmaceutical design.

## Background

Multiple HIV-1 proteins recruit host cullin-based E3 ligase components to promote ubiquitination and degradation of factors that restrict viral replication [1]. Human APOBEC3 (A3) cytidine deaminases consist of a family of potent inhibitors of HIV [2-4]. Viral infectivity factor (Vif) is a 192 amino acid (23 kDa) accessory protein that is conserved amongst all lentiviruses with the exception of equine infectious anemia virus [5]. Vif recruits an E3 ubiquitin ligase complex, which promotes A3 polyubiquitination and subsequent degradation via the proteasome [6-9]. The Vif-based viral E3 ubiquitin ligase complex consists of Vif, Cullin 5 (Cul5), Elongins B/C (Elo B/C), and a RING-box protein (Rbx) [6]. In addition, a new binding partner, core binding factor beta (CBF-β) was recently discovered to bind Vif, function in the Vif-Cul5 ligase and regulate A3G/F suppression [10-16].

In order to hijack the Cul5-E3 ligase complex, Vif mimics cellular protein motifs that are responsible for recruiting ubiquitin ligase components [7,17-23]. Many of these motifs have been found within Vif’s carboxyl terminus. Vif contains a conserved SOCS box domain, including the BC Box motif (residues 144–155) and the cullin box (residues 158–173) [7,17]. The BC Box serves as the primary attachment point between Vif and Elo B/C [6,7,17]. Structural and biophysical data indicates that there is a second weaker interaction between the semi-conserved Vif cullin box and Elo B/C, but this has been found not to be required for Elo B/C and Vif interaction [22,24]. Rather, this weak interaction has been demonstrated to position the cullin box, particularly the PPLP motif, for Cul5-Vif interaction [22,24]. Importantly, a single amino acid substitution in the highly conserved lentiviral Vif SOCS box reduces the ability of Vif to block virion packaging of A3G and to fully suppress the antiviral activity of A3G [7,17]. In addition to the role of the cullin box, a Vif zinc binding domain, ^108^HX_5_-CX_17-18_-CX_3-5_-H^139^ (HCCH), has been reported to mediate the primary interaction with Cul5 [18-20,23]. As expected, mutation of either the HCCH domain or the cullin box severely inhibits Cul5 binding and A3G degradation [18-20,22-24].

While the C-terminus has been implicated in binding several E3 ubiquitin ligase components, several discontinuous Vif residues in the amino-terminus have been reported to be necessary for A3G/F interaction [25-30]. However, reports have also demonstrated that the C-terminal cullin box mediates contact with A3G [31,32]. In addition, CBF-β binds an amino-terminal motif of Vif; Vif tryptophan residues 21 and 38 are key mediators of this interaction [11]. We have demonstrated that silencing CBF-β expression in mammalian cells severely suppresses Vif-Cul5 formation [11]. In addition, recombinant CBF-β increases Vif solubility, *in vitro*[13]. These two points suggest that CBF-β is important for the structural integrity of Vif molecules.

While both CBF-β and APOBEC3 proteins interact with Vif at the N-terminus, it is unclear whether Vif makes contact with E3 ubiquitin ligase components in the N-terminus. Our lab and others have reported amino acids in the N-terminus that are important for A3G/F degradation, yet do not mediate interaction with A3G/F by co-immunoprecipitation [25,26]. We also showed that a N-terminal truncated (amino acids 99–192) Vif couldn’t precipitate endogenous Cul5 in 293 T cells, although others have demonstrated that a N-terminal truncated Vif has a high binding affinity for Cul5, *in vitro*[11,33]. However, a group recently reported Cul5 binds to full length Vif with greater affinity when compared to a C-terminal half fragment containing both the HCCH domain and cullin box [12]. Taken together, these reports suggest that Cul5 may interact with or require additional Vif residues in the N-terminus. Here, we report that, indeed, Cul5 recruitment requires a Vif N-terminal motif (^25^VXHXMY^30^). More importantly, this motif is required specifically for Cul5, but not Elo B/C or CBF-β, to interact with full length Vif in mammalian cells as well as in recombinant form. Using isothermal titration calorimetry, we demonstrate that a single point N-terminal Vif mutant has a severely reduced binding affinity for Cul5 compared to wild-type Vif. Vif N-terminal mutants that disrupted Cul5 formation were also less efficient at degrading A3G/F and restoring HIV infectivity in the presence of A3G. Interestingly, Vif mutants that do not bind Cul5, including Vif His108Ala, are structurally different from Vif wild-type and mutants that do bind Cul5. Thus, it is unclear whether these Vif residues are responsible for direct interaction with Cul5.

## Results

### N-terminal motif in full length Vif is important for Cul5 interaction, *in vitro*

We have previously reported that a Vif truncation mutant containing residues 99–192 is insufficient for binding to Cul5 in mammalian cells [11], but we were curious whether a longer N-terminal truncated Vif protein or a C-terminal truncated Vif protein may bind to Cul5. Interestingly, neither the Vif N-terminal (residues 1–91) nor C-terminal (residues 92–192) protein could pull down endogenous Cul5 by co-immunoprecipitation (Additional file 1: Figure S1). Thus, we posited that Cul5 must require both residues in the N- and C-terminus of Vif in order to form an interaction. To address this question, we decided to construct several Vif N-terminal mutant plasmids, altering residues to alanine in a predicted alpha helix that contains several residues important for APOBEC3 degradation

First, we confirmed whether each mutant was capable of forming a complex with CBF-β, Elo B/C and Cul5 in *E. coli*. Wild-type or mutant Vif was co-expressed with Cul5, Elo B/C, and 6X-His-tagged CBF-β (amino acids 1–140) in NiCo21(DE3) competent *E. coli* (NEB) at 23C with 0.2 mM IPTG. After 24 hrs, cells were harvested, lysed by sonication, and clarified by centrifugation. His-tagged CBF-β and interacting partners were pulled down from supernatants using Ni-NTA affinity beads (Qiagen).

**Figure 1:**
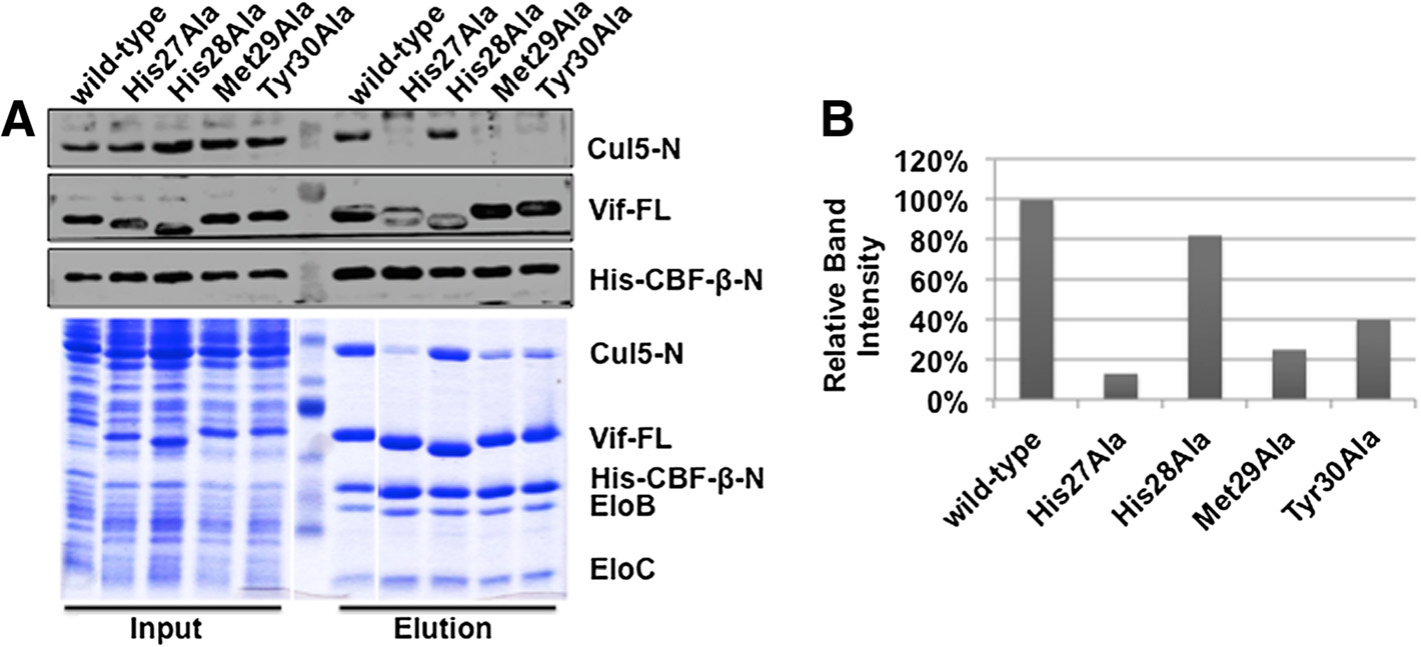
**Vif N-terminal amino acids are responsible for Cul5 interaction, *in vitro*.** Vif wild-type and single alanine mutants were co-expressed with N-terminal Cul5, N-terminal 6X-His-CBF-β (residues 1–140), and Elo B/C. Next, the complex was pulled down using nickel affinity purification. **A)** While Vif wild-type and H28A mutant pull down Cul5 efficiently, H27A, M29A and Y30A mutants are unable to bind Cul5 efficiently. **B)** Quantitative measurement of the Cul5 band intensity was performed indicating the relative amount of Cul5 bound to Vif wild-type and mutant protein complexes. FL – full length, N-terminal – amino-terminus, 6X-His – 6X histidine tag.

Vif mutants His27Ala, Met29Ala, and Tyr30Ala contributed to Vif-Cul5 interaction, *in vitro,* both reducing Cul5 binding by greater than 60% (Figure 1A, 1B). Interestingly, His28 minimally contributed to the interaction between Vif and Cul5, only reducing Cul5 binding by approximately 20% (Figure 1A, 1B). Taken together, the experimental evidence suggests that an N-terminal motif regulates the Vif-Cul5 interaction using recombinant protein. We wanted to further explore this possibility in mammalian cells.

### Vif N-terminal motif binds Cul5 in mammalian cells and is required for APOBEC3 degradation and restoring HIV infectivity

**Figure 2:**
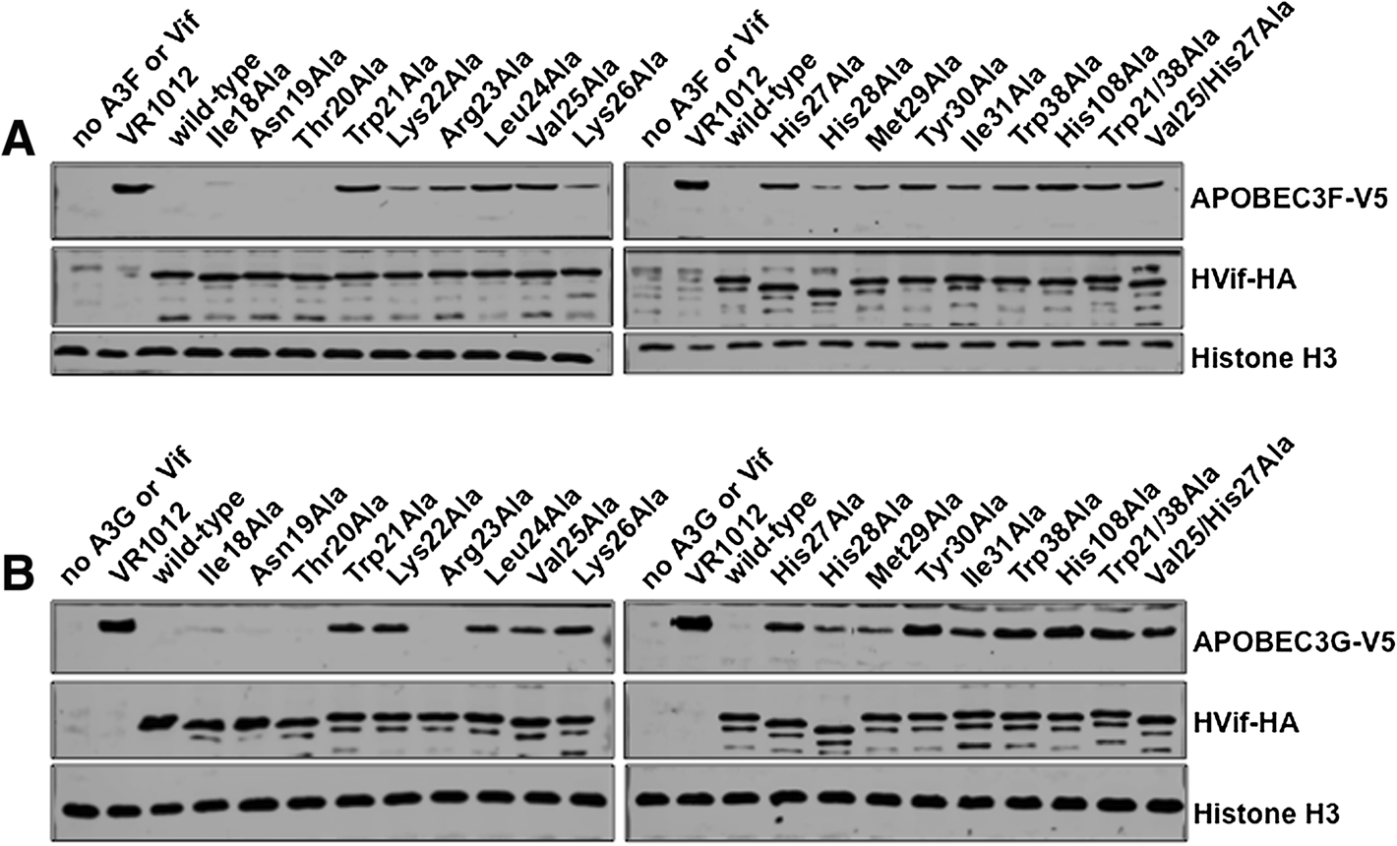
**Several Vif N-terminal mutants are unable to degrade APOBEC3G and APOBEC3F.** Vif wild-type and mutant proteins along with A3G or A3F were overexpressed in HEK 293 T cells. Two days post-transfection, cells were lysed and proteins were separated by SDS-PAGE and visualized by western blotting. **A)** APOBEC3F-V5 and **B)** APOBEC3G-V5 are efficiently degraded by wild-type Vif, but not by several Vif N-terminal mutants in HEK 293 T cells.

While we were confident in our experimental evidence demonstrating that there were amino acids in the Vif N-terminus that are responsible for Cul5 interaction, *in vitro*, we wanted to explore the importance of this motif in mammalian cells. We decided to assess the importance of additional residues within this alpha helix in mediating APOBEC3 degradation. Several single point mutant constructs were created replacing amino acid residues 18–31 with alanine. Next, HEK 293 T cells were co-transfected with plasmids containing either A3G or A3F with wild-type Vif, mutant Vif or empty vector. As expected, wild-type Vif efficiently mediated A3G as well as A3F degradation compared to control vector (Figure 2A, 2B). Importantly, mutation of Vif residues, His27, Met29, and Tyr30 led to a reduced capacity for Vif to degrade both A3G and A3F. Vif mutants, L24A, V25A, and to a lesser extent I31A also inhibited Vif’s ability to degrade both A3G and A3F (Figure 2A, 2B). In agreement with our prior observation that His28 was unimportant for Vif-Cul5 formation, the Vif-H28A mutant along with Vif-R23A were both capable of inducing A3G and A3F degradation similarly to wild-type Vif (Figure 2A, 2B). Finally, lysine 22 and 26 were important for degradation of A3G not A3F (Figure 2A, 2B).

**Figure 3:**
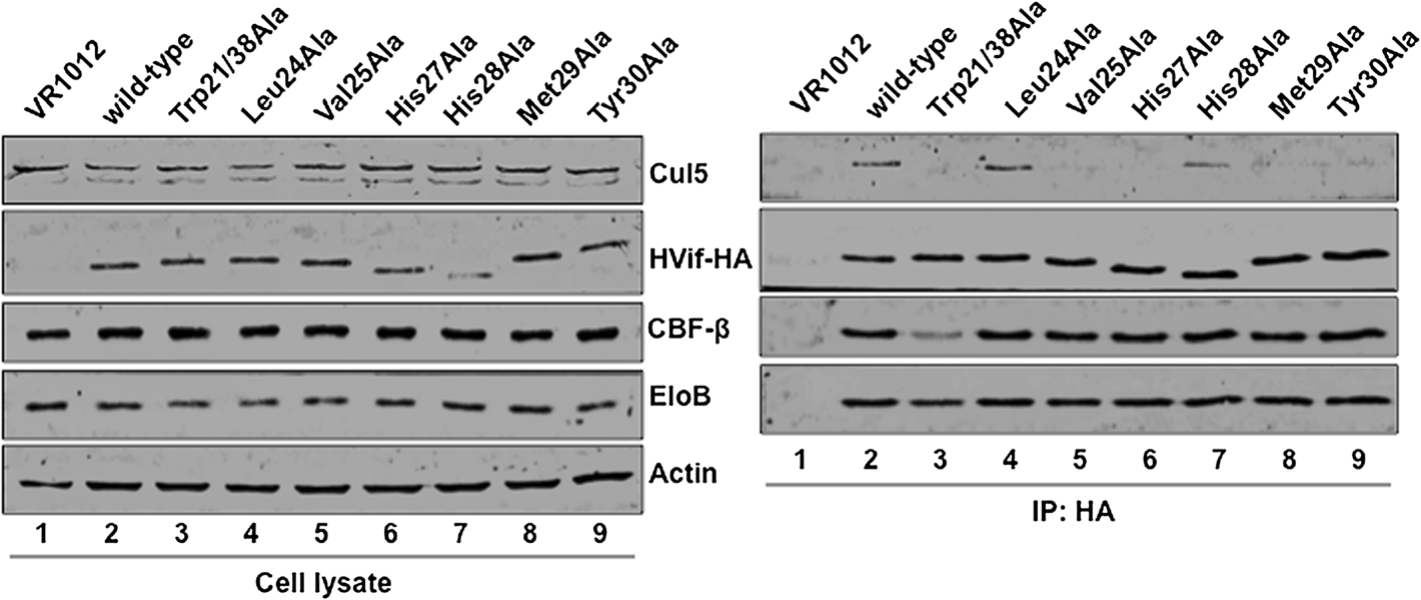
**Select Vif N-terminal mutants have a reduced ability to bind Cul5 in mammalian cells.** HA-tagged Vif wild-type and mutant proteins along with CBF-β were overexpressed in HEK 293 T cells. Two days post-transfection, cells were lysed and cleared lysate was mixed with anti-HA matrix affinity beads for 4-8 hrs. Incubated beads were washed several times followed by elution of bound proteins. Select Vif N-terminal mutants (V25A, H27A, M29A, and Y30A) that do not efficiently degrade A3G and A3F have a reduced ability to co-precipitate Cul5; however, CBF-β and Elo B/C can still bind Vif.

**Figure 4:**
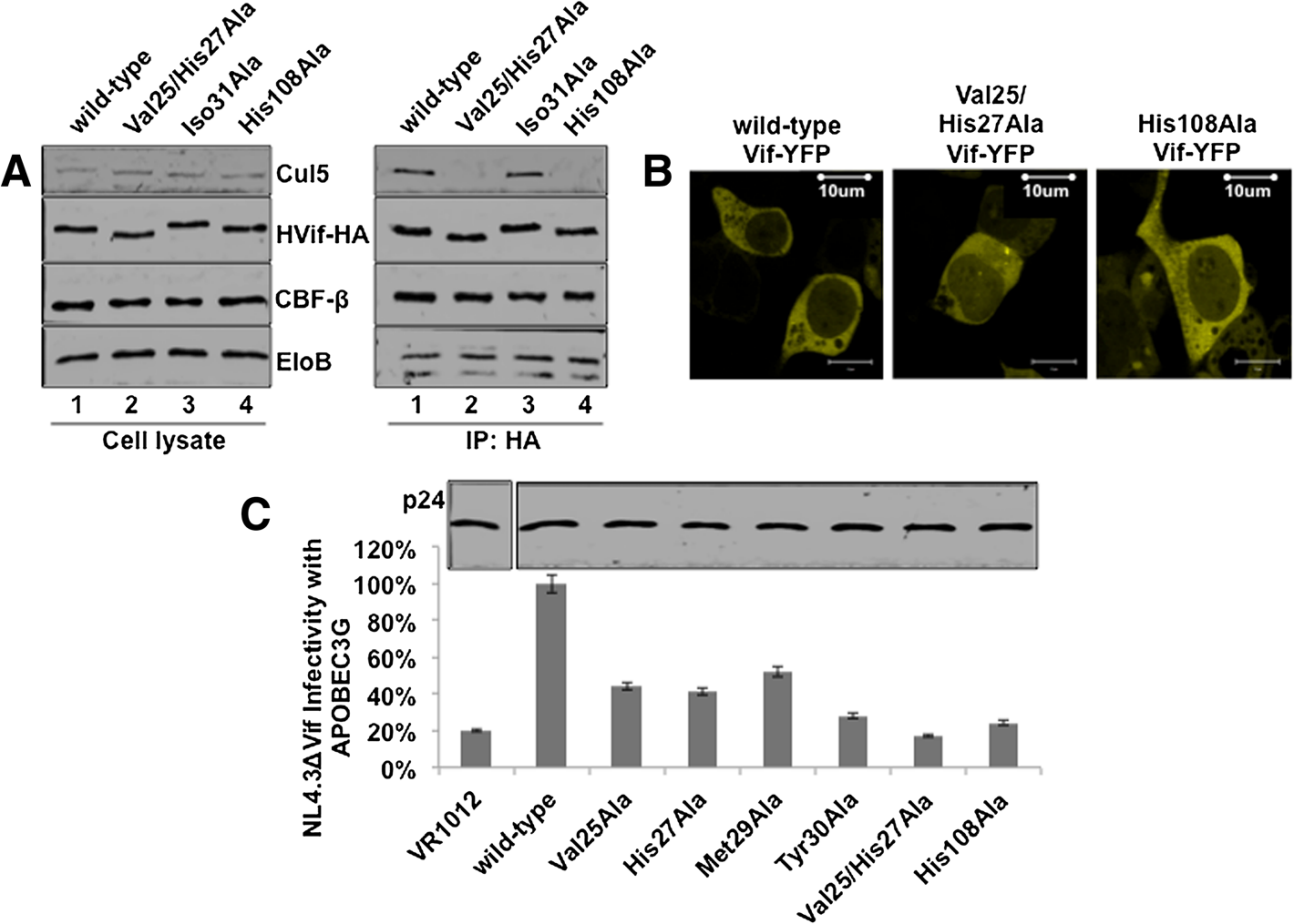
**Vif N-terminal mutants localize to cytoplasm, but are inefficient at restoring HIV infectivity.** HA-tagged Vif wild-type and mutant proteins along with CBF-β were overexpressed in HEK 293 T cells. Two days post-transfection, cells were lysed and cleared lysate was mixed with anti-HA matrix affinity beads for 4-8 hrs. Incubated beads were washed several times followed by elution of bound proteins. **A)** Select Vif N-terminal mutants that do not efficiently degrade A3G and A3F have a reduced ability to co-precipitate Cul5; however, CBF-β and Elo B/C can still bind Vif. **B)** Plasmids (Vif-YFP 2 ug and CBF-β 0.5 ug) were transfected into 293 T cells using Lipofectamine 2000 (Invitrogen), according to the manufacturer’s protocol. Cells were visualized at 25°C using a Zeiss LSM510-Meta confocal imaging system. Imaging demonstrates that the Vif double mutant V25/H27A and single mutant H108A localize to the cytoplasm of the cell similar to wild-type. **C)** Vif wild-type and mutant containing virus were produced and used to infect MAGI cells. Infected cells were stained using X-gal. The histogram demonstrates that Cul5-binding deficient Vif mutants were inefficient at restoring HIV infectivity in the presence of A3G. Error bars represent the standard error from triplicate experiments. Capsid p24 levels are shown in the western blot.

To investigate which Vif N-terminal amino acids interact with Cul5, we immunoprecipitated HA-tagged single point mutants that were inefficient at degrading both A3G and A3F. Vif wild-type and mutant plasmids were transfected into 293 T cells. In order to enhance Vif-Cul5 binding as well as Vif and endogenous Cul5 expression, CBF-β was overexpressed and the proteasome inhibitor, MG132, was added to the culture media, respectively. HA-tagged Vif along with bound proteins were immunoprecipitated using anti-HA affinity matrix beads (Roche). As expected, wild-type Vif co-precipitated with Cul5 as well as CBF-β and Elo B (Figure 3, lane 2). Consistent with our previous report [11], Vif residues, Trp21 and Trp38, were important for CBF-β as well as Cul5 binding (Figure 3, lane 3). Also as expected, Vif mutant, H108A was incapable of binding Cul5, however maintained its ability to bind both Elo B/C and CBF-β (Figure 4A, lane 4). Vif mutants, H27A, M29A, and Y30A, but not H28A were unable to co-precipitate endogenous Cul5, consistent with *in vitro* results (Figure 3). An additional Vif residue, Val25, also contributed to Cul5 binding (Figure 3). Interestingly, Vif L24A and I31A, which were unable to mediate both A3G and A3F degradation, still bound Cul5 similar to wild-type Vif (Figures 3, 4A). Furthermore, while a Vif double mutant V25H27A was unable to bind Cul5, it was still able to bind CBF-β and Elo B/C (Figure 4A) as well as localize to the cytoplasm of the cell similar to wild-type (Figure 4B), suggesting that the N-terminal mutants are structurally stable and available for Cul5 cytoplasmic assembly.

Next, we investigated whether N-terminal mutants that were unable to degrade A3G were restricted in restoring HIV infectivity in the presence of A3G. 293 T cells were transfected with Vif-deficient pNL4.3 plasmid along with plasmids overexpressing A3G and wild-type or N-terminal mutant Vif proteins. Virus was harvested from the supernatant after 36 h and used to infect MAGI cells as previously described [11]. As expected, Vif N-terminal mutants had a reduced ability to restore HIV infectivity in the presence of APOBEC3G (Figure 4C). Single alanine mutants, V25A, H27A, M29A, and Y30A were at least 40% less efficient compared with wild-type at restoring HIV infectivity. Furthermore, double mutant V25H27A was as inefficient as the H108A mutant compared to wild-type Vif.

### Binding affinity between Cul5 and Vif N-terminal single point mutant is significantly reduced

Recently, a group reported that the binding affinity between Cul5 and full length Vif complexed with Elo B/C and CBF-β was enhanced by nearly 80-fold compared to a N-terminal truncated Vif-Elo B/C complex [12]. Since we had observed reduced binding between Vif mutants and Cul5 by pull-down assays, we postulated that single point mutants in the N-terminus of Vif would reduce the binding affinity for Cul5. To test our hypothesis, we performed ITC experiments to directly measure the affinity between recombinant Cul5 and wild-type Vif as well as N- and C- terminal mutants (V25A and H108A) complexed with CBF-β and Elo B/C.

**Figure 5:**
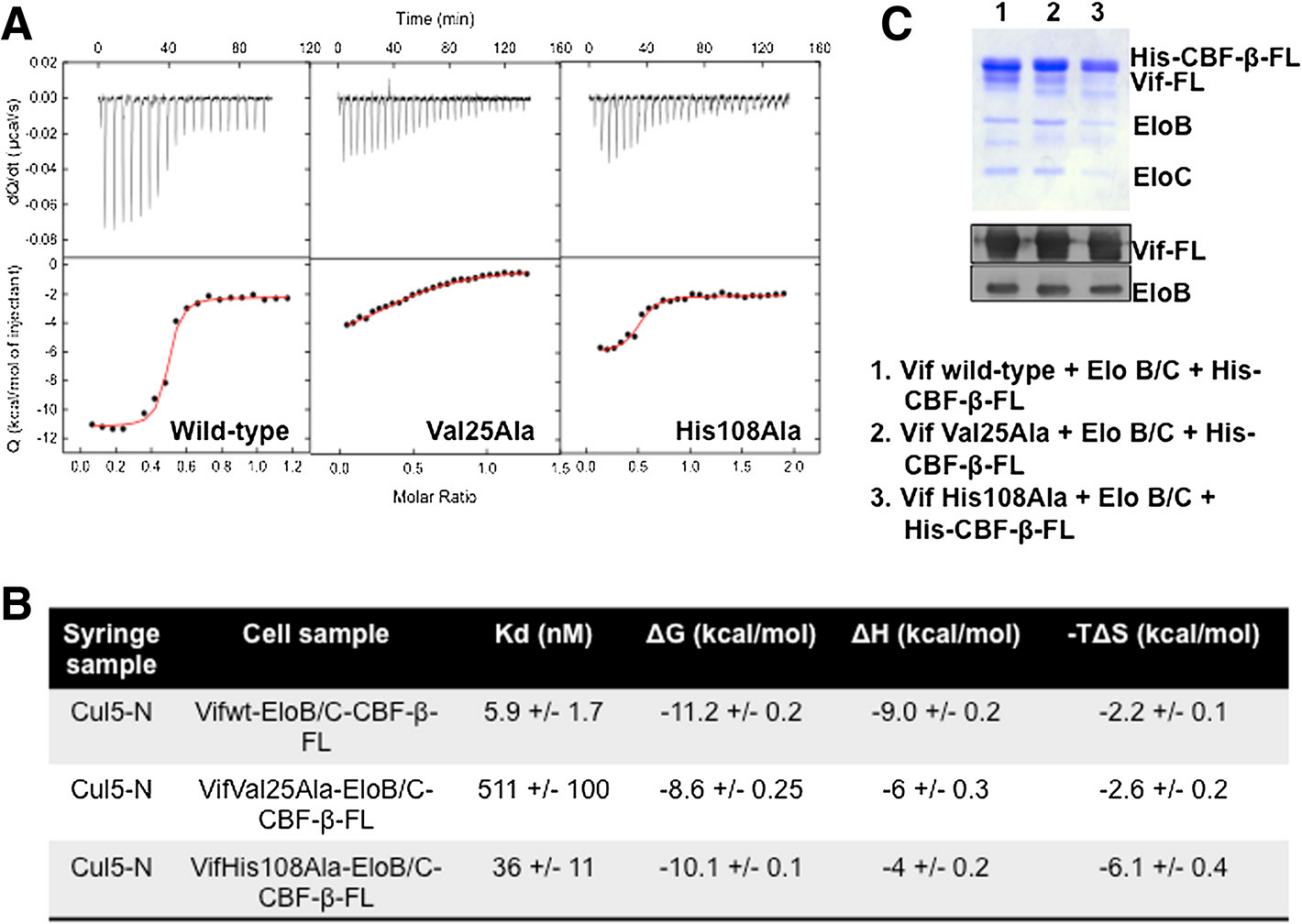
**Recombinant Vif mutants have a lower Cul5 binding affinity compared to wild-type Vif.** Isothermal titration calorimetric analyses of the interaction between Cul5 and Vif wild-type and mutant complexes. **A)** and **B)** Representative ITC isotherm and table for Vif wild-type and mutants demonstrating a lower affinity between mutant Vif and Cul5 compared with Vif wild-type. **C)** Vif complex samples run on SDS-PAGE gel and visualized by coomassie stain and western blot. FL – full length.

Vif complexes including his-tagged full length CBF-β and Elo B/C in addition to GST-Cul5 were separately overexpressed in E. coli and purified using affinity and size exclusion chromatography, as previously described [13]. ITC experiments were employed to determine the binding thermodynamics between Vif wild-type and mutant complexes. Wild-type or mutant Vif-CBF-β-Elo B/C complexes in the calorimetric cell were titrated by stepwise additions of Cul5. Cul5 bound to the wild-type Vif-CBF-β-Elo B/C complex with an affinity of 5.9 nM, which corresponded to change in Gibbs free energy of -11.2 kcal/mol at 25°C. The contributions from the enthalpy (ΔH) and entropy (-TΔS) to Gibbs free energy were -9.0 and -2.2 kcal/mol, respectively (Figure 5A, 5B). The values are consistent with the previously published measurements by Salter *et al.*[12]. Additionally, Vif N-terminal mutant complexes that were still capable of binding to Cul5 (i.e. L24A and H28A) bound to Cul5 with an affinity similar to wild-type (Additional file 2: Figure S2).

Next, a change from valine to alanine at position 25 in the N-terminal mutant Vif V25A complex resulted in a loss of enthalpic interactions of -3 kcal/mol, which translates to a 90-fold loss in binding affinity (Kd = 511 nM) (Figure 5A,B). Furthermore, the N-terminal mutant Vif H27A complex was similar to the V25A complex with a binding affinity of 660 nM (Additional file 2: Figure S2). Intriguingly, the Vif C-terminal point mutant (H108A), which is part of the reported HCCH motif, only reduced the binding affinity of Cul5 for Vif by 6-fold (Kd = 36 nM) (Figure 5A,B). The loss in enthalpic interactions were, in fact, larger for the binding to the H108A mutant (ΔH = -4.0 kcal/mol) but because the entropy contribution was more favorable (TΔS = -6.1 kcal/mol) and partially compensated the loss in enthalpy, the overall binding affinity was reduced to a lesser extent than for the binding to the V25A mutant (Figure 5A,B).

### Vif mutant complexes are structurally different from wild-type

**Figure 6:**
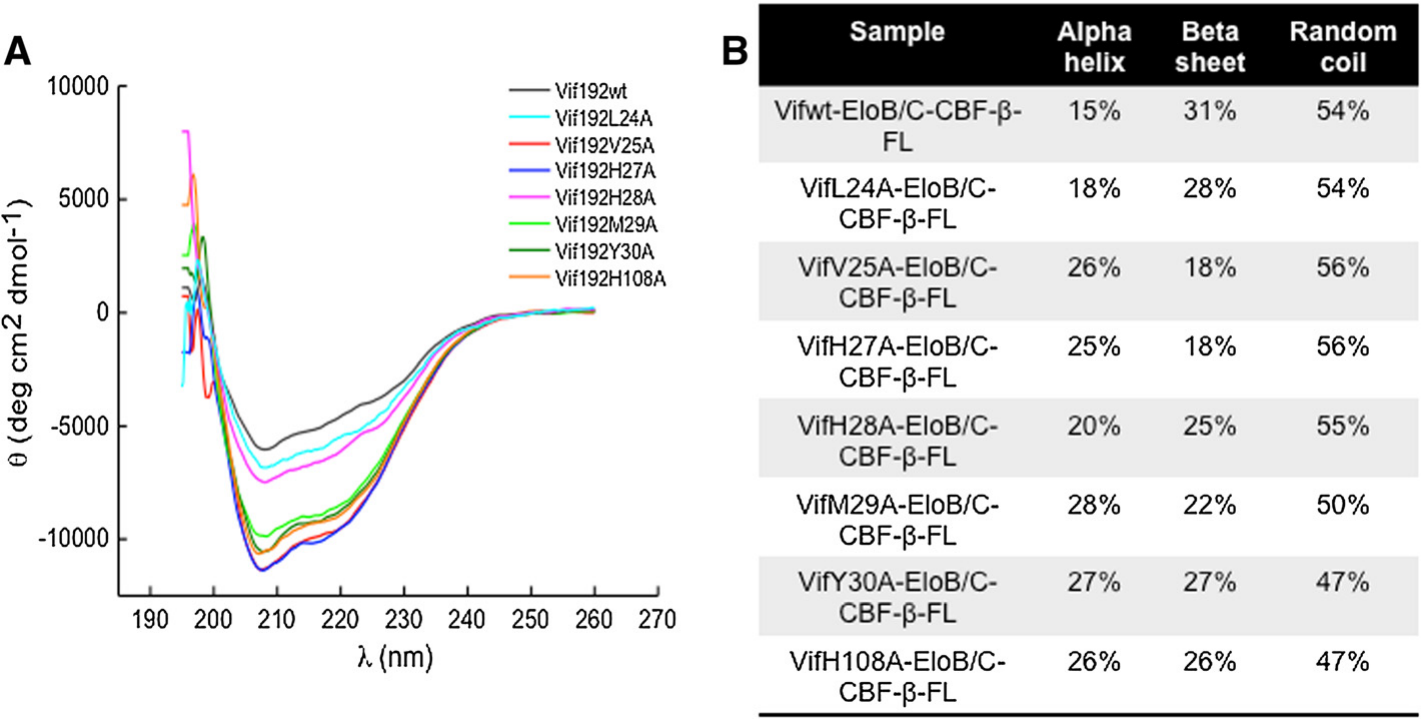
**CD spectroscopy analysis reveals that Vif mutant complexes are structurally different from wild-type.** Purified Vif complexes including 6X-His-CBF-β (residues 1–182) and Elo B/C were purified by nickel affinity and size exclusion chromatography. Each complex was analyzed by circular dichroism spectroscopy. **A)** CD spectra for Vif wild-type and mutant complexes showed a distinction in the minima at 208 and 222 for mutant complexes that do not bind Cul5, suggesting these mutants have more alpha helical structure. **B)** Spectra analysis reveals differences between wild-type and mutant complex secondary structure and confirms that the mutants that do not bind Cul5 have a higher percentage of alpha helical structures; however, the percentage of beta-sheet structures is reduced.

To determine if the mutant complexes were structurally similar to wild-type Vif complexes, the complexes were all analyzed by circular dichroism spectroscopy (Figure 6A). Vif complexes (0.4 mg/mL) were measured at room temperature in PBS with TCEP (0.25 mM). Subsequent analysis of the CD spectra with the Dichroweb [34,35] analysis program suggested that all of the N-terminal mutant Vif complexes that don’t bind Cul5 (i.e. V25A, H27A, M29A, and Y30A) had a higher percentage of alpha helical structure and lower percentage of beta sheet structure (Figure 6B). Surprisingly, the CD spectra for the Vif H108A C-terminal mutant complex suggested that our control mutant is also structurally different than wild-type (Figure 6B). Furthermore, Vif complexes that did bind Cul5 were very similar in structure (Figure 6B). Additionally, gel filtration chromatography results appear to be consistent with this change in secondary structure. All mutant complexes, which were unable to bind Cul5, eluted slower from a Superdex 200 (GE Healthcare) size-exclusion column (Additional file 3: Figure S3).

## Discussion

**Figure 7:**
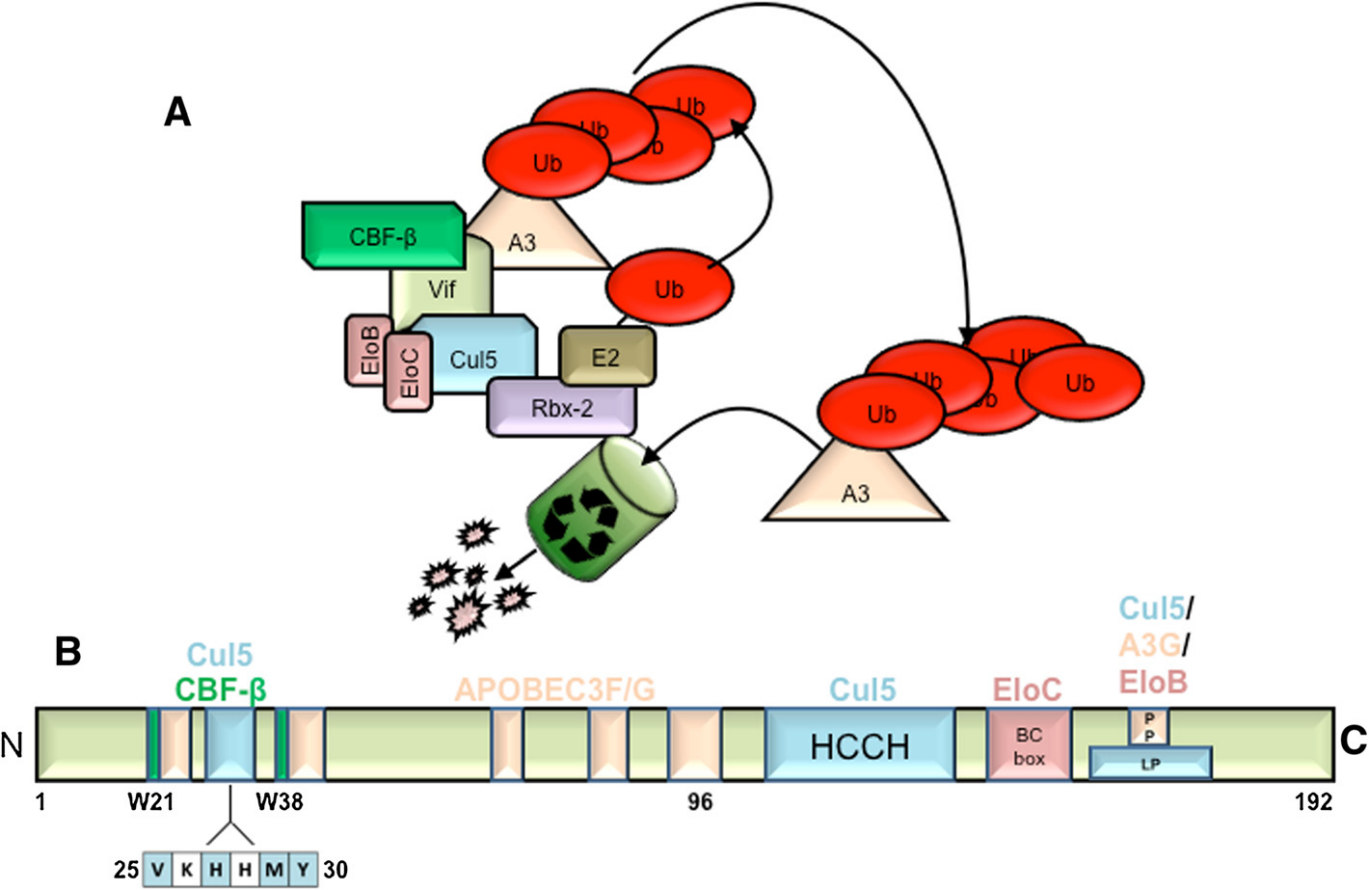
**Model for Vif-E3 ligase complex and schematic diagram of Vif residues required for E3 ligase recruitment. A)** Model for Vif-mediated ubiquitination and degradation of A3 molecules via recruitment and assembly of host E3 ligase components. **B)** Updated schematic diagram of Vif binding motifs, including the Vif N-terminal motif required for efficient Cul5 binding.

Here, we report an enhanced understanding of the molecular interactions between the HIV-1 Vif protein and the host E3 ubiquitin ligase scaffold protein, Cul5. Data from multiple reports have contributed to a model in which Vif recruits an E3 ubiquitin ligase complex, including Cul5, Elo B/C, Rbx, and co-factor CBF-β to promote A3 polyubiquitination and subsequent degradation via the proteasome (Figure 7A) [6-16]. Previously, our group and others demonstrated the importance of Vif residues Val25, His27, and Tyr30 for APOBEC3 suppression [25-27]. For the first time, we demonstrate through multiple lines of experimental evidence that Met29 contributes to A3G/F suppression and that an N-terminal motif (^25^VXHXMY^30^) in the Vif polypeptide is required for Cul5 interaction (Figure 7B), thus providing a rationale for its essential role in suppressing APOBEC3 proteins. Based on our previous and current data as well as recently published data by Wang X. *et al.*[36], the recruitment of Cul5 to the Vif E3 ubiquitin ligase can be summarized in the following steps: (1) full length Vif is unbound to Cul5 in the absence of CBF-β and Elo B/C; (2) Vif binds Elo B/C at its C-terminus followed by CBF-β at its N-terminus, inducing structural changes at both termini; (3) once Vif is bound to both CBF-β and Elo B/C, Cul5 binds to Vif, requiring both residues in the N- and C-terminus of the protein to assemble a functional ubiquitin ligase. We have demonstrated previously that Vif does not require Elo B/C [6] or CBF-β [11] in order to bind A3G. In addition, A3G binding is not necessary for full length Vif to bind Cul5 [13]. Thus, Vif may recruit A3G prior to or after Cul5 incorporation.

The once difficult task of producing full-length soluble Vif for *in vitro* experimental analyses have been overcome with the discovery of one of Vif’s binding partners, CBF-β [10,11,13,16]. We have exploited this recent finding in order to successfully co-express and purify recombinant full-length Vif with known interacting partners CBF-β, Elo B/C, and Cul5. Through pull-down assays, we initially observed a weak interaction between several Vif N-terminal mutants (i.e. H27A, M29A, and Y30A) and Cul5. These mutants specifically reduced the interaction between Vif and Cul5, not affecting its interaction with other interacting partners, CBF-β and Elo B/C. Interestingly, mutation of His28 had minimal effect on Vif-Cul5 formation. Furthermore, these Vif mutants were required for Cul5 interaction in mammalian cells as well as for efficient A3G/A3F degradation. Val25 was also found to be critical for association with Cul5. Isothermal titration calorimetry analyses revealed that both Vif V25A and H27A mutants had a reduced affinity for Cul5 (>90-fold reduction) compared to wild-type Vif, while C-terminal mutant H108A reduced the affinity 6-fold. It is plausible that these residues affect Vif tertiary structure, thus affecting Vif-Cul5 interaction. However, we observed that both N- and C-terminal binding partners, CBF-β and Elo B/C still bind to all of these mutants, suggesting that the Vif single alanine point mutants remain in a structural state similar to the native conformation. Yet, we found that the structure of the protein complex does change when either N-terminal (i.e. V25A, H27A, M29A, and Y30A) or C-terminal (i.e. H108A) residues are altered to alanine using both circular dichroism spectroscopy and size-exclusion chromatography. Thus, the changes in N-terminal and C-terminal amino acids can alter the structure of Vif such that CBF-β and Elo B/C still bind, yet precludes Cul5 binding.

Previous reports suggested that the ^23^S/RLV^25^ motif was important for APOBEC3 suppression [6,7,25,26]. Our results demonstrate that Leu24 and Val25 are most critical in this motif; Arg23 played no or a minor role in A3G and A3F suppression, respectively. Given that residue ^23^S/R is highly conserved between HIV and SIV molecules, it is unclear whether this residue plays a role independent of APOBEC3 degradation. In addition, Vif H108A bound to Cul5 with reduced binding affinity when compared to wild-type; however, the affinity was stronger than the Vif V25A mutant. Comparatively, single mutation of this histidine residue to alanine results in a marked decrease in Cul5 interaction by immunoprecipitation analysis in mammalian cells (Figure 4A). Furthermore, reports have demonstrated using ITC that the Vif C-terminal domain is sufficient to recruit Cul5, although full length Vif had a greater binding affinity for Cul5 compared to C-terminal domain fragments [12,33]. It is plausible that the V25A mutant complex fell apart during the ITC measurement; however, it appeared intact similar to wild-type throughout the purification protocol (Figure 5C). Next, while Vif L24A had a diminished ability to suppress both A3G and A3F, this mutant could still bind to Cul5 as well as CBF-β and Elo B/C. Dang *et al.* reported that mutation of this leucine residue also had no effect on A3G/F binding [26]. It is intriguing to think that there is a yet to be discovered Vif binding partner that interacts with this residue and is necessary for efficient A3G/F suppression. Alternatively, mutation of the hydrophobic residue may disrupt Vif conformation in a manner that interferes with A3 ubiquitination.

His27/28 was previously reported to contribute to A3G suppression [27]. Here we show that His27, but not His28, contributes to both A3G and A3F suppression as well as mediates contact with Cul5. It has not been ruled out whether His28 may play a role in mediating the interaction and suppression of other APOBEC3 proteins; however, previous reports have observed that Vif H28A mutant HIV clones grow as well as wild-type in the presence of A3G or A3F [37,38].

Remarkably, the discovered Vif N-terminal motif that facilitates binding with Cul5 is in close proximity to amino acids that mediate binding to its substrate molecule, APOBEC3 as well as its regulator, CBF-β. A3G and A3F bind discontinuous motifs in the N-terminus and some reports have suggested that it makes contact with a C-terminal motif [25-28,30-32,38]. We have demonstrated previously that Trp21 and Trp38 are important for both CBF-β and Cul5 binding and we confirm the importance of these residues in this report (Figures 2A, 2B, and 3) [11]. Tyr30 was previously suggested to be important for both RNA binding as well as A3G binding and suppression [25,39]. In addition, substitution of histidine for tyrosine at position 30 in Vif was found to correlate with a reduced ability to transmit the virus from mother to child, supporting the importance of this residue for Vif function [40]. Tyr30 was critical for suppression of A3G, although we also observed reduced suppression of A3F. To our surprise, we observed that this residue also mediates Cul5 interaction. It is unclear whether A3F may also interact with this residue. However, it appears that this residue doesn’t entirely disrupt A3F interaction because we do not observe complete recovery of A3F levels as we do with A3G levels in the presence of this mutant compared to empty vector. How a single Vif residue interacts with both A3G and Cul5 is a mystery, although this is not the first time that a Vif residue has been implicated in binding more than one protein [22,31-33]. The secondary structural changes that we observed for Vif mutant complexes may explain this phenomenon. In addition, we can appreciate that Vif is an extremely complex macromolecule and understanding precisely how it interacts with all of its partner host proteins will require solving its 3-dimensional crystal structure.

There are currently over 30 approved HIV drugs, which target only a few of the 15 HIV proteins encoded in its genome. However, problems related to drug failure, emergence of drug-resistant variants, and treatment-related adverse consequences persist. Thus, the expansion of anti-HIV therapies requires new targets. The discovery of novel inhibitors that combat additional HIV proteins depends upon the understanding of how viral proteins bind cellular factors to increase viral fitness. The precise role of each of the Vif domains in mediating interaction with cellular proteins requires further study. The insights to be gained from exploring these possible interfaces between Vif and the E3 ligase complex components have the potential to contribute to the design of novel HIV inhibitors.

## Conclusion

We have identified a Vif N-terminal motif that is required for binding to Cul5 and proposed an updated model for Vif recruitment of E3 ligase components critical for APOBEC3 suppression and successful HIV replication. Thus, the Vif-Cul5 interaction requires three discontinuous regions, located in both the N- and C-termini of the Vif polypeptide. Future studies should focus on determining whether additional Vif N-terminal residues are important for Cul5 association, whether additional residues in Cul5 are responsible for Vif interaction, and ultimately solving the Vif-E3 ligase complex tertiary structure to increase the likelihood of discovering an effective Vif-E3 inhibitor.

## Methods

### Plasmid construction

*E. coli constructs*: Human Cul5 ([13]) and NL4.3 Vif coding sequences were cloned into the pET-Duet plasmid using 5′ NcoI/3′ EcoRI (MCS1) and 5′NdeI/3′ XhoI (MCS2) restriction sites, respectively to create Cul5/Vif pET-Duet constructs. All point mutants of Vif were created by site-directed mutagenesis. Elongin B and Elongin C (residues 17 to 112) in the pACYC-Duet plasmid were a gift from Alex Bullock. The genes for Elongins B and C were subcloned into the pCDF-Duet vector. CBF-β isoform 2 (residues 1–182) and truncated CBF-β (residues 1–140) from human were cloned into MCS1 of pRSF-Duet ([13]) to create 6X-His-tagged-CBF-β-Full length(-FL) and CBF-β-N-terminus (-N). wild-type and mutants of Vif were subcloned into MCS2 of pRSF-Duet using 5′NdeI/3′ XhoI restriction sites with CBF-β to create his-CBF-β/Vif pRSF-Duet.

*Mammalian cell constructs*: HVif-HA was constructed by PCR amplifying codon optimized Vif from pcDNA-HVif and cloning the product into VR1012 plasmid via EcoRI and BamHI restriction sites. The following primers were used to create HVifHA: forward 5′ – CTCTCTGAATTCATGGAGAACCGGTGG – 3′ and reverse 5′ - ATGGATCCCTACGCGTAATCTGGGACGTCGTAAGGGTAGTGTCCATTCATTG – 3′ (HA). To generate yellow fluorescent protein (YFP) epitope-tagged NL4-3 viral infectivity factor (Vif), pCDNA HVif wild-type and mutant constructs were used to PCR amplify the *vif* coding region and cloned into the BamH1 and EcoRI sites of pEYFP-N1.

### Protein expression and purification

Plasmids were transformed or co-transformed into *Escherichia coli* NiCo21(DE3) cells(New England Biolabs C2529H) according to manufacturer’s protocol. Cells were incubated for two hours at 37°C and plated on media with appropriate antibiotic selection marker. If more than one plasmid was transformed, cells were centrifuged at 5,000 rpm for 5 min and then all cells were plated. Plates were incubated at 37°C overnight and single colonies were chosen for protein production. Cells were grown to an OD of 0.8-1 at 37°C, cooled to 23°C, and induced overnight at 23°C with 0.2 mM isopropyl-D-thiogalactopyranoside (IPTG). Harvested cells containing his-CBF-β-Vif-Elo B/C complexes were lysed in lysis buffer (1X PBS, 0.25 mM TCEP, 30 mM imidazole), sonicated, and centrifuged at 10,000 g for 20 min. Nickel affinity purification – soluble supernatant was added to Ni-NTA beads (Invitrogen) and incubated at room temperature for 2 h. Beads with bound protein were washed 6X with wash buffer (1XPBS, 0.25 mM TCEP, 40 mM imidazole). Bound protein was eluted with elution buffer (1X PBS, 0.25 mM TCEP and 250 mM imidazole). Gel filtration using a Superdex 200 column (GE Healthcare) was utilized to remove trace contaminants. Harvested cells containing Cul5-NTD (residues 1 to 393 with two point mutations, V341R, L345D, and) were lysed in lysis buffer (1X PBS and 0.25 mM TCEP). The supernatant was transferred to glutathione-Sepharose 4B beads (GE Healthcare) for purification. The GST tag was then removed using PreScission protease (GE Healthcare) in lysis buffer at 4°C for 36 h. Cul5-NTD was subsequently purified by gel filtration.

### Transfection, co-immunoprecipitation, and infectivity assay

HEK293T cells were maintained at 37C, 5% CO2 in DMEM (Invitrogen, catalog 11995073) with added 10% fetal bovine serum (Sigma, catalog F4135). Vif-HA and A3-V5 plasmids were complexed with PEI-Max at a 2.5:1 ratio in Opti-Mem (Invitrogen, catalog 31985070) buffer for 30 min. Cells were transfected with plasmid: PEI complexes and harvested 48 h later, washed with PBS and lysed in lysis buffer (50 mM Tris, 75 mM NaCl, 0.1% NP-40 and Complete Protease Inhibitor Cocktail Tablet (Roche, catalog 04693159001) (pH 7.4)) at 4°C for 15 min, followed by centrifugation at 10,000 × *g* for 20 min. HA immunoprecipitation was carried out by mixing soluble lysates of transfected cells from a 10 cm dish with 40 uL anti-HA affinity matrix beads (Roche, catalog 11815016001) and incubating the mixture at 4°C for 3 h. The samples were washed six times with wash buffer (20 mM Tris, 50 mM NaCl, 0.1 mM EDTA and 0.05% Tween-20 (pH 7.5)). Bound protein was eluted with elution buffer (100 mM glycine-HCl, pH 2.5). The eluted protein was analyzed by SDS-PAGE and immunoblotting with appropriate antibodies. Infectivity (MAGI) assay was performed as previously described [11].

### Live cell confocal imaging

Plasmids (Vif-YFP 2 ug and CBFB 0.5 ug) were transfected into 293 T cells using Lipofectamine 2000 (Invitrogen), according to the manufacturer’s protocol. Cells were visualized at 25°C using a Zeiss LSM510-Meta confocal imaging system equipped with four argon lasers (458, 477, 488, and 514 nm lines), two HeNe lasers (542 and 633 nm), and one diode laser (405 nm). All images were acquired from a 100X objective, and image analysis and manipulation was performed using Zen 2009 software.

### Immunoblot analysis

Proteins were separated by SDS-PAGE, followed by transfer to nitrocellulose membrane (Bio-Rad). After blocking with PBS-buffered saline-Tween 20 containing 5% BSA for 20 min at room temperature, membranes were incubated with a specific antibody overnight at 4°C. After three washes with PBS-buffered saline-Tween 20, the membranes were stained with an alkaline phosphatase-conjugated secondary antibody (1:3,000, Jackson Immunoresearch) for 2 h at room temperature. After three washes with PBS-buffered saline-Tween 20, the membranes were incubated in development buffer containing 5-bromo-4-chloro-3′-indolylphosphate (BCIP) and nitro-blue tetrazolium (NBT) substrate (Sigma). The antibodies used in this study were specific for: Vif (the AIDS Research Reagents Program, catalog 2221), Cul5 (Santa Cruz Biotechnology Inc., catalog sc-13014), CBF-β (Abcam, catalog ab11921), Elo B (Santa Cruz Biotechnology, Inc, catalog sc-11447), Elo C (BD Transduction Laboratories, catalog 610760), HA (Invitrogen, catalog 715500), and V5 (Invitrogen, catalog R96025).

### Isothermal titration calorimetry

ITC experiments were performed using a VP- ITC microcalorimeter from MicroCal/GE Healthcare (Northampton, MA, USA). Each protein or protein complex was purified by a Glutathione or Ni-NTA affinity protocol followed by gel filtration in PBS, pH 7.4, with 0.25 mM TCEP. Titrations were conducted by adding Cul5 in steps of 10 μL every 300 s to the calorimetric cell (volume ~1.4 mL) containing wild-type or mutant Vif-CBF-β-Elo B/C complex. The concentrations of Cul5 and the Vif-CBF-β-Elo B/C complex were 25 and 2.5 μM, respectively. Saturation was reached in 20–28 injections. All experiments were conducted at 25°C. The heat evolved upon each injection of Cul5 was obtained from the integral of the calorimetric signal. The heat associated with binding to Vif-CBF-β-Elo B/C complex in the cell was obtained by subtracting the heat of dilution from the heat of reaction. The individual heats were plotted against the molar ratio, and the enthalpy change (ΔH) and association constant (K_a_ = 1/K_d_) were obtained by nonlinear regression of the data.

### Circular dichroism spectroscopy

CD experiments were conducted using a Jasco J-710 spectropolarimeter. Wavelength scans were performed with 0.4 mg/mL Vif complexes prepared in buffer containing PBS with 0.25 mM TCEP in a 0.1 cm cuvette in a water-jacketed cell. Spectra were averaged over 3 consecutive scans collected from 195 to 260 nm. The individual scans were recorded using a scan rate of 20 nm/min, a bandwidth of 1 nm and a response time of 2 s per point. Buffer scans were accumulated and subtracted from the sample scans and the mean residue ellipticity was computed. The temperature was kept constant at 25°C. Spectral analysis was performed using the Dichroweb online analysis program [34,35]. Initial and final wavelengths were 260 and 195 nm, respectively, in wavelength steps of 0.2 nm. Analysis was performed using the K2D algorithm.

## Abbreviations

HIV-1: Human immunodeficiency virus type 1; Vif: Viral infectivity factor; WT: Wild-type; A3G: APOBEC3G; A3F: APOBEC3F; Cul5: Cullin 5; RBX2: RING-box protein 2; Elo B/C: Elongin B/C; CBF-β: Core binding factor beta; E2: Ubiquitin conjugating enzyme; E3: Ubiquitin ligase; HCCH: Vif zinc binding motif, ^108^HX_5_-CX_17-18_-CX_3-5_-H^139^; FL: Full length; PBS: Phosphate-buffered saline; SDS-PAGE: Sodium dodecyl sulfate-polyacrylamide gel electrophoresis; (ITC): Isothermal titration calorimetry; (CD): Circular dichroism.

## Competing interests

The authors declare that they have no competing interests.

## Author’s contributions

SLE, AS, QG, XH, and XZ conducted the experiments and analyzed the data. SLE conceived the study, and SLE, XFY, EF supervised the project. SLE wrote the manuscript. All authors read, edited, and approved the final manuscript.

## Supplementary Material

Additional file 1: Figure S1 Cul5 does not bind N-terminal or C-terminal half of Vif in mammalian cells. HA-tagged Vif 1–91 and Vif 92–192 truncation mutants were over-expressed in 293 T cells, harvested and proteins analyzed by co-immunoprecipitation and SDS-PAGE. Wild-type Vif can co-immunoprecipitate endogenous Cul5, CBF-β, and Elo B/C; however, Vif 1–91 and Vif 92–192 cannot pull down Cul5. Click here for file

Additional file 2: Figure S2 Recombinant Vif mutants have a lower Cul5 binding affinity compared to wild-type Vif. 5A) and 5B) Representative ITC isotherm and table for Vif wild-type and mutants (Leu24Ala, His27Ala, and His28Ala) demonstrating that His27Ala has a lower affinity between Cul5 compared with Vif wild-type. However, mutants Leu24Ala and His28Ala have a similar affinity for Cul5 compared with Vif wild-type. Click here for file

Additional file 3: Figure S3 Vif mutants that don’t bind Cul5 elute later from size-exclusion column. Vif wild-type and mutant complex samples were purified by gel filtration chromatography. Wild-type and mutants of Vif that bind to Cul5 eluted together; however, Vif mutants that do not bind Cul5 eluted later from the column. Click here for file
