# Supplementary figures and images for: HIV-1 Vif N-terminal Motif is required for recruitment of Cul5 to Suppress APOBEC3
Source: Retrovirology. 2014 Jan 14;11:4. doi: 10.1186/1742-4690-11-4 (PMC3937519; doi:10.1186/1742-4690-11-4)

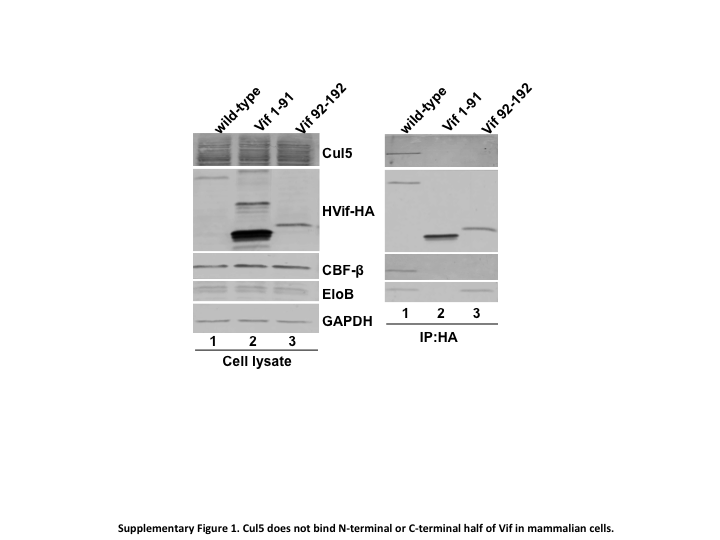

Supplement: Additional file 1: Figure S1 — Cul5 does not bind N-terminal or C-terminal half of Vif in mammalian cells. HA-tagged Vif 1–91 and Vif 92–192 truncation mutants were over-expressed in 293 T cells, harvested and proteins analyzed by co-immunoprecipitation and SDS-PAGE. Wild-type Vif can co-immunoprecipitate endogenous Cul5, CBF-β, and Elo B/C; however, Vif 1–91 and Vif 92–192 cannot pull down Cul5. [file 1742-4690-11-4-S1.png]

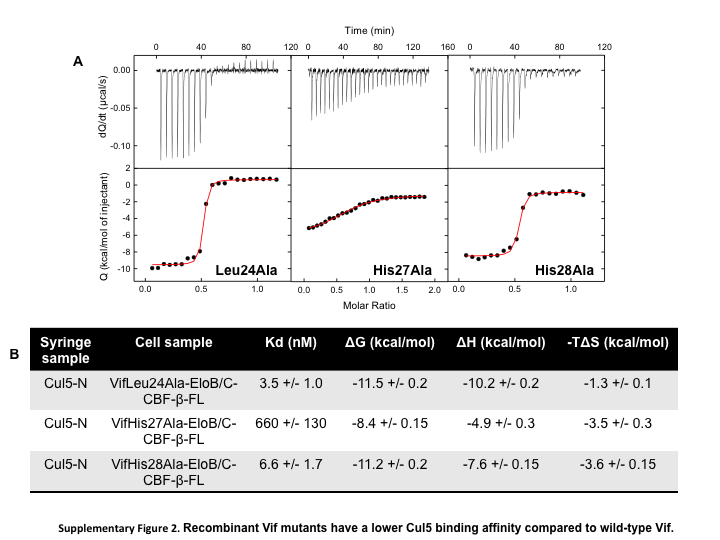

Supplement: Additional file 2: Figure S2 — Recombinant Vif mutants have a lower Cul5 binding affinity compared to wild-type Vif. 5A) and 5B) Representative ITC isotherm and table for Vif wild-type and mutants (Leu24Ala, His27Ala, and His28Ala) demonstrating that His27Ala has a lower affinity between Cul5 compared with Vif wild-type. However, mutants Leu24Ala and His28Ala have a similar affinity for Cul5 compared with Vif wild-type. [file 1742-4690-11-4-S2.png]

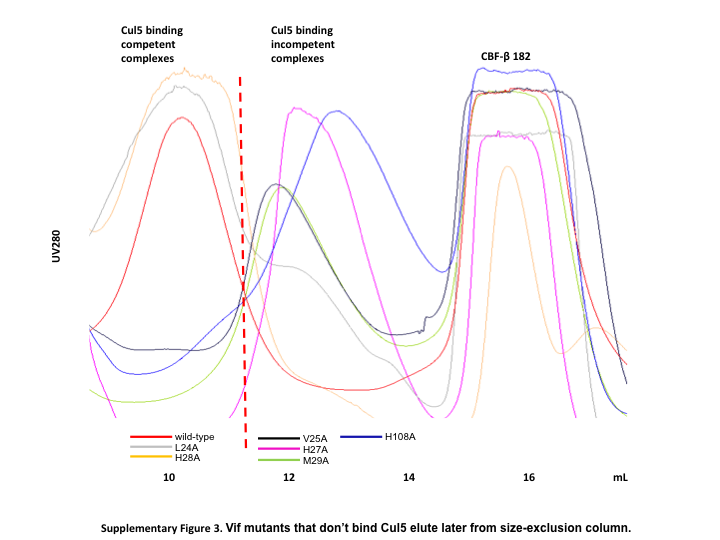

Supplement: Additional file 3: Figure S3 — Vif mutants that don’t bind Cul5 elute later from size-exclusion column. Vif wild-type and mutant complex samples were purified by gel filtration chromatography. Wild-type and mutants of Vif that bind to Cul5 eluted together; however, Vif mutants that do not bind Cul5 eluted later from the column. [file 1742-4690-11-4-S3.png]
